# Diagnostic Validation of the Screening Corneal Objective Risk of Ectasia Analyzer Evaluated by Swept Source Optical Coherence Tomography for Keratoconus in an Asian Population

**DOI:** 10.3390/bioengineering10111335

**Published:** 2023-11-20

**Authors:** Kookyoung Kim, Kyungmin Koh, Seongjun Lee, Yongwoo Lee

**Affiliations:** 1 Nuri Eye Hospital, Daejeon 35233, Republic of Korea; 2 Kim’s Eye Hospital, Seoul 07301, Republic of Korea; 3 Departement of Ophthalmology, Kangwon National University Hopsital, Kangwon National University School of Medicine, Chuncheon, Gangwon 24289, Republic of Korea

**Keywords:** corneal topography, keratoconus, SCORE Analyzer, swept-source OCT

## Abstract

We aimed to investigate the diagnostic accuracy of Screening Corneal Objective Risk of Ectasia (SCORE) Analyzer software using ANTERION, a swept-source optical coherence tomography device, for keratoconus diagnosis in an Asian population. A total of 151 eyes of 151 patients were included in this retrospective study as follows: 60, 45, and 46 keratoconus, keratoconus suspects, and normal control eyes, respectively. Parameters in the SCORE calculation, including six indices, were compared for the three groups. The receiver operating characteristic curve analysis and cut-off value were estimated to assess the diagnostic ability to differentiate keratoconus and keratoconus suspect eyes from the normal group. The SCORE value and six indices were significantly correlated—“AntK max” (R = 0.864), “AntK oppoK” (R = 0.866), “Ant inf supK” (R = 0.943), “Ant irre 3mm” (R = 0.741), “post elevation at the thinnest point” (R = 0.943), and “minimum corneal thickness” (R = −0.750). The SCORE value showed high explanatory power (98.1%), sensitivity of 81.9%, and specificity of 78.3% (cut-off value: 0.25) in diagnosing normal eyes from the keratoconus suspect and keratoconus eyes. The SCORE Analyzer was found to be valid and consistent, showing good sensitivity and specificity for keratoconus detection in an Asian population.

## 1. Introduction

Keratoconus is a progressive disease characterized by thinning and outward bulging of the cornea, thereby affecting vision. Corneal topography is the only useful examination for determining corneal changes in the early stage of keratoconus [1]. It is a technique that measures the shape and curvature of the cornea and can be used to detect and diagnose keratoconus using various methods. However, there are currently no diagnostic tools available that can fully detect subclinical keratoconus. Individual parameters obtained by corneal topography—such as keratometry > 47 diopters (D) (defined as a “steep” K) [2], abnormal inferior keratometry minus superior keratometry (I-S) values [3], and a thin cornea (<500 µm) [4]—are not necessarily indicative of keratoconus and when considered alone may generate false positives. Consequently, keratoconus cannot be diagnosed with only one parameter, and multiple diagnostic criteria—such as KISA% [5], Klyce [6], keratoconus prediction index (KPI) [7], and Belin/Ambrosio enhanced ectasia display (BAD) [8], which use corneal thickness, corneal curvature, and corneal elevation as measured by corneal topography—have been developed and used [9].

The Screening Corneal Objective Risk of Ectasia (SCORE) Analyzer software was first developed by Dr. Damien and Dr. Saad to provide clinicians with a unique number to rate the ectasia susceptibility of myopic eyes using linear discriminant analysis with ORB scan corneal topography data [10]. They developed the new SCORE Analyzer using corneal parameters measured by ANTERION (Heidelberg Engineering, Heidelberg, Germany), a swept-source optical coherence tomography (SS-OCT) device. The parameters generated in the algorithm of the SCORE Analyzer and the eyes used for its validation were based on data from white individuals. Consequently, it is necessary to investigate the effectiveness and diagnostic accuracy of the SCORE Analyzer in an Asian population as there are inherent differences among eyes of different ethnicities; these include the risk of myopia, risk, incidence, and progression of keratoconus, and variations in corneal hysteresis and central corneal thickness. These differences can be attributed to variations in genetic makeup [11,12,13,14]. Differences in corneal topographic parameters between Asian and white ethnic groups have also been described [15].

Therefore, in this study, we aimed to investigate the accuracy and clinical validation of the SCORE Analyzer in diagnosing keratoconus in Korean eyes.

## 2. Materials and Methods

We retrospectively reviewed the medical records of patients who underwent SS-OCT at Kim’s Eye Hospital and Nuri Eye Hospital from February 2020 to May 2023. The study protocol was approved by the Institutional Review Board (IRB number: KNUH-2023-08-008) of Kangwon National University Hospital, Chuncheon-si, Korea, and the study was conducted in accordance with the tenets of the Declaration of Helsinki.

Overall, 60 eyes of patients with keratoconus, 45 eyes of keratoconus suspect patients, and 46 eyes with normal corneas were included in this study. In the case of keratoconus suspect or keratoconus in both eyes, only one eye was included randomly. Accordingly, the data of 151 patients were analyzed in this study.

The diagnosis of keratoconus and suspected was based on clinical examination and patterns based on topography. A diagnosis of keratoconus was confirmed when one or more of the following clinical outcomes were observed; (1) anterior protruding of the cornea, (2) stromal thinning (3) Fleischer ring or Vogt striae on slit lamp inspection, (4) asymmetric bowtie map, (5) abnormal steepening of the cornea on topographic images. Amsler–Krumeich class I, II, and III keratoconus without treatment history were included in the study. Corneal opacity or hydrops that affected corneal topography measurements were excluded.

Keratoconus suspect was defined as the contralateral eye of a clinical presenting keratoconus eye with the following characteristics; (1) no clinical findings (keratometric, retinoscopic, or biomicroscopic) of keratoconus; (2) inferior–superior asymmetry and/or bowtie pattern with skewed radial axes (SRAX), detected via Scheimpflug topography; and (3) no history of contact lens wear, ocular surgery, or trauma [16].

The normal control group was defined as individuals with no ocular disease, no previous ocular surgery, and no irregular finding on the corneal topography. The exclusion criteria were patients with glaucoma, suspicion of glaucoma, corneal scarring, pregnancy, infective keratitis, or an underlying systemic autoimmune disease.

Furthermore, for all patients, a complete ophthalmologic examination was performed including mydriatic fundus examination. The SS-OCT examination was performed by a skilled operator. The measurement results confirmed that if all three groups corresponded to the acquisition quality parameters of “Motion”, “Fixation”, and “Tear film and lid”, they were all qualified as “pass’’, and if any “pass” qualification failed to appear, repeated examination was performed.

The SCORE Analyzer, developed based on ORB scan topography, incorporates 12 corneal indices, including pachymetry, location of the thinnest point, difference between the inferior and superior parts, and irregularity [17]. Six values of these parameters were integrated into the ANTERION SCORE for analysis.

1. “Ant. Kmax”: The maximum K value of the anterior corneal surface.
2. “Ant. Kmax—opposite K (AntK_oppoK)”: The difference between the maximum K value of the anterior corneal surface and the K value measured opposite the Kmax position.
3. “Ant. inf.—sup. K mean (Ant_inf_supK)”: The difference between the inferior mean anterior corneal axial curvature and the superior mean anterior corneal axial curvature.
4. “Ant. irregularity (Ant_irre_3 mm)”: The irregularity of the axial curvature of the anterior corneal surface at the central 3 mm ring.
5. “Posterior elevation of thinnest point (post_ele_thin)”: The elevation of the posterior corneal surface at the location of the thinnest point with the best fit sphere as a reference surface.
6. “Thinnest point (A_Cor_thin)”: The thinnest position of the cornea.

SCORE values of 0 and higher were assigned to the color range of yellow to red. The SCORE bar visually locates the SCORE value on a color-scale bar with a value ranging from −4 to 20, which is not adjustable.

### Statistical Analysis

The data were analyzed using the Statistical Package for Social Sciences (SPSS) Statistics for Windows (version 26.0; IBM Corp., Armonk, NY, USA). The sample size was determined using MedCalc software (version 22.013; MedCalc Software Ltd., Ostende, Belgium) to achieve a statistical power exceeding 80%, based on previously published results [10,18].

The Shapiro–Wilk test was performed to evaluate the normality of the data. The Baseline data and anterior segment indices were compared using the Kruskal–Wallis test. Group data were compared using the Mann–Whitney test and Bonferroni’s adjustment. Comparisons of categorical data were performed using the Chi-square test. Receiver operating characteristic (ROC) curves were designed to determine the overall prediction accuracy of each parameter, as indicated by the area under the curve (AUC).

We determined the cutoff value to distinguish between keratoconus and the normal group by Youden’s J statistic.

Pearson’s correlation coefficient was used to analyze the correlation between the SCORE value and the six indices. Stepwise multivariate regression analysis was constructed with the SCORE value as the dependent variables and the six indices as covariates. Statistical significance was set at *p* < 0.05.

## 3. Results 

Table 1 presents the demographic data of the included patients; no statistically significant difference was found between the three groups except for the astigmatism (*p* < 0.001). All topography parameters showed significant differences in all three groups except for the horizontal position of the thinnest point (Table 1).

Table 2 presents the SCORE value and the six indices in the three groups, expressed as mean ± standard deviation. All parameters were significantly different between the three groups (*p* < 0.05). In the post hoc test, for the minimal corneal thickness value, no significant difference was observed between the normal group and the keratoconus suspect group (*p* = 0.319); however, all other values were significantly different.

According to Pearson’s test, the SCORE value significantly correlated with all six indices: AntK max (R = 0.864), AntK oppoK (R = 0.866), Ant inf supK (R = 0.943), Ant irre 3 mm (R = 0.741), post_ele_thin (R = 0.943), and A_Cor_thinthe minimal corneal thickness (R = −0.750) (all *p* < 0.05).

The results of the stepwise multivariate linear regression analysis for the SCORE value as the dependent variable with six indices are summarized in Table 3. The SCORE value showed high explanatory power (98.1%) when composed of six indices (SCORE value = −9.267 + 0.344 × AntK_max + 0.880 × AntK_oppoK + 1.082 × Ant_inf_supK − 0.939 × Ant_irre_3 mm + 0.09 × post_ele_thin − 0.014 × A_Cor_thin, adjusted R^2^ = 0.981; *p* < 0.001).

**Figure 1:**
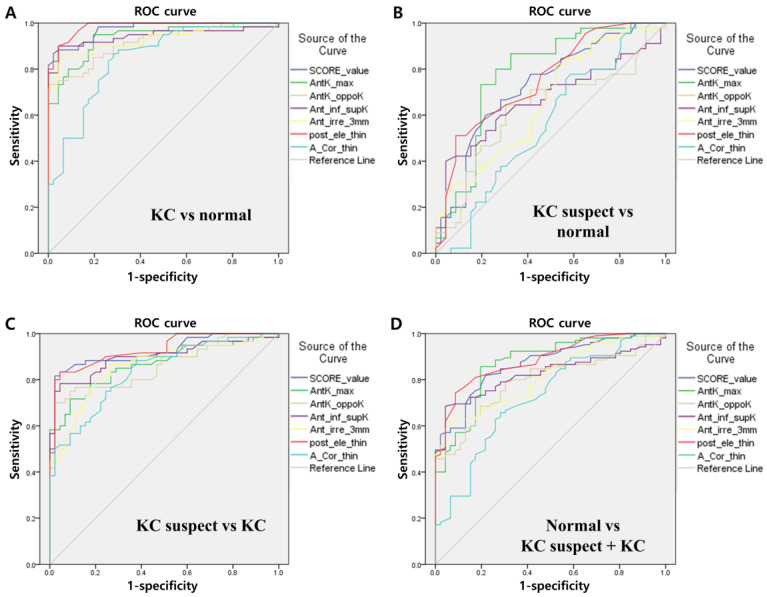
Receiver operator characteristic (ROC) curve analysis of the SCORE value and the six indices for keratoconus eyes versus keratoconus suspect or normal eyes. (**A**) ROC curves for keratoconus eyes versus normal eyes. (**B**) ROC curves for normal eyes versus keratoconus suspect eyes. (**C**) ROC curves for keratoconus suspect eyes versus keratoconus eyes. (**D**) ROC curves for normal eyes versus keratoconus and keratoconus suspect eyes. KC: keratoconus; “Ant. Kmax”: The maximum K value of the anterior corneal surface; “Ant. Kmax—opposite K (AntK_oppoK)”: The difference between the maximum K value of the anterior corneal surface and the K value measured opposite the Kmax position; “Ant. inf.—sup. K mean (Ant_inf_supK)”: The difference between the inferior mean anterior corneal axial curvature and the superior mean anterior corneal axial curvature; “Ant. irregularity (Ant_irre_3 mm)”: The irregularity of the axial curvature of the anterior corneal surface at the central 3 mm ring; “Posterior elevation of thinnest point (post_ele_thin)”: The elevation of the posterior corneal surface at the location of the thinnest point with the best fit sphere as a reference surface; “Thinnest point (A_Cor_thin)”: The thinnest point of the cornea.

Figure 1 shows the results of the ROC curve analyses of eyes with keratoconus and keratoconus suspect with the normal group using SCORE Analyzer. The area under the ROC curve (AUROC) of the SCORE value for diagnosing keratoconus from the normal group was 0.974. Among the six indices, the posterior elevation at the thinnest point showed the highest AUROC value (0.985) as a single variable. Relatively, the minimal corneal thickness showed a low AUROC value (0.847). The AUROC of the SCORE value and six indices for diagnosing keratoconus suspect eyes from normal eyes were relatively low compared to other comparison groups. In most comparison groups, the posterior elevation at the thinnest point showed a high AUROC value as a single variable.

Table 4 shows the ROC curve analysis, optimal cut-off point, and sensitivity and specificity of the optimal cutoff point for each variable tested in the keratoconus, normal, and suspected keratoconus group. For diagnosing the keratoconus group from the normal group, the sensitivity was 86.8% and the specificity was 97.8% with SCORE (cut-off value: 2.4). For diagnosing the keratoconus suspect and the keratoconus groups from the normal group, the sensitivity was 81.9% and the specificity was 78.3% with SCORE (cut-off value: 0.25).

## 4. Discussion

Herein, we aimed to investigate the accuracy and clinical validation of the SCORE Analyzer in diagnosing keratoconus in a Korean population. We found that the AUROC for SCORE values measured using the SS-OCT ranged from 0.729 to 0.974 for discriminating normal cornea from keratoconus or keratoconus suspect eyes.

The new SS-OCT anterior segment device (Anterion^®^, Heidelberg Engineering, Heidelberg, Germany) has been clinically proven to be useful in the evaluation of anterior segment [19,20], cataract [21], and glaucoma diseases [22]. The SS-OCT method is considered more advantageous for the topographic and tomographic evaluation of the ectatic cornea than the conventional Placido type or Scheimpflug method, owing to its higher number of radial scans, shorter scan times, and real-time eye tracking system, which detects eye movement and the corneal vertex [23].

Keratoconus should be diagnosed early owing to the following reasons. First, keratoconus is a progressive disease usually diagnosed during the second and third decades of life. Presently, attempts to halt the progression of keratoconus are made with corneal collagen cross-linking, which ultimately reduces the need for corneal transplantation [24]. Second, young people in Asia with myopia generally prefer corneal laser refractive surgery for vision correction; therefore, iatrogenic ectasia needs to be prevented through precise preoperative evaluation. Currently, the most commonly used diagnostic tool for the high-risk group of early keratoconus is corneal topography [6,25]. However, the measurement principle is different for each corneal topography examination, and the reference values used to diagnose keratoconus are also diverse. Consequently, it is important to confirm the validity and accuracy of the reference values in actual clinical practice before applying new corneal topography or diagnostic criteria.

The first SCORE Analyzer was developed by Saad and Gatinel based on values measured by the Orbscan IIz corneal topography system (Bausch + Lomb TechnoLas, Munich, Germany) and designed to detect Forme Fruste keratoconus corneas (FFKC) [10].

A recent study by Saad and Gatinel [18] found that combining anterior and posterior curvature variables along with pachymetric data obtained from SS-OCT allows automated detection of early keratoconus with reasonable accuracy (87% and 99.5%, respectively). The SCORE Analyzer is based on a linear regression analysis that constructs a set of linear functions of variables, known as discriminant functions. Unlike Orbscan IIz, ANTERION is a multimodal imaging platform that utilizes the power of high-resolution SS-OCT images to provide corneal topography and tomography. SS-OCT is faster and sensitive; it was used to measure the anterior and posterior shape of the cornea, as well as the cross-sectional tomographic images [26,27].

Hickson-Curran et al. showed the differences in corneal topographic parameters between Asian and white ethnic groups [15]. In our study, the overall average WTW was about 12.08 mm, similar to the values of using OCT with an Asian population in a previous study [28], with smaller WTW values compared to Westerners. Because previous studies on the SCORE Analyzer were mostly from Western countries, it is necessary to explore whether these diagnostic criteria can be applied to Asian populations as well. This is the first study to investigate the diagnostic accuracy and clinical validation of the SCORE Analyzer for keratoconus using ANTERION in an Asian population.

The SCORE value and the six indices used for its calculation demonstrated different correlations. “Ant. inf.—sup. K mean” and “Posterior elevation of thinnest point” showed the highest correlation, and “Ant. irregularity (3 mm)” and “Thinnest point” showed a relatively low correlation. The stepwise multivariate linear regression analysis showed a very high explanatory power of 98.1% between the SCORE value and the six indices measured for an Asian population.

A previous study verifying the validity of the keratoconus diagnosis using the SCORE Analyzer of Orbscan corneal topography showed a sensitivity of 92% and a specificity of 96% [29]; they classified 183 Westerners as “normal” or “at risk for LASIK” using ORB corneal topography, and the SCORE Analyzer was also used to classify the corneas based on a SCORE value of 0. In the present study, 0 was not specified as a cut-off value, but a new cut-off value was presented considering sensitivity and specificity for each comparison group. The cut-off value for distinguishing between normal and keratoconus eyes was 2.4, with a 0.974 AUROC value. It appeared as a number higher than 0, and the sensitivity and specificity were 0.868 and 0.978, respectively. The cut-off value for distinguishing between normal eyes and keratoconus suspect was −0.05, close to 0, and the AUROC value (0.729) was relatively lower than that of the other comparison groups. The cut-off value for the distinction between the normal group and the keratoconus suspect and keratoconus groups was 0.25, and the AUROC value was 0.869. An AUC of 0.90–1 represents excellent discrimination ability, and an AUC of 0.80–0.90, 0.70–0.80, 0.60–0.70, and 0.50–0.60 indicates good, fair, poor, and very poor discrimination ability, respectively [30]. In the present study, the SCORE value measured using the SS-OCT ANTERION had AUROC values ranging from 0.729 to 0.974 for discriminating normal eyes from keratoconus or keratoconus suspect eyes, thereby providing clinically useful data for diagnosing keratoconus.

The SCORE Analyzer of the ANTERION indicates a higher likelihood of ectatic change when the score value is 0 or greater. In this study, the cut-off value to distinguish between normal eyes and keratoconus suspect eyes was close to zero (−0.05). In addition, the cut-off value for distinguishing suspected keratoconus from keratoconus eyes was 4.45. Therefore, using SCORE Analyzer, when an abnormal corneal topography shows a score of at least 2.4 or higher, keratoconus can be diagnosed. Also, among the six indices of the SCORE value, high “Ant. inf.—sup. K mean” and “Posterior elevation of thinnest point” values were more meaningful in representing keratoconus changes. Among the six indices, the “posterior elevation of thinnest point” showed the highest AUROC value in most of the comparison groups. This result is similar to that of other previous studies, which reported that elevations were the best parameters for the keratoconus diagnosis [31,32]. Our recent study also showed high sensitivity and specificity for discriminating normal from keratoconus eyes using the posterior corneal elevation based on the best-fit toric ellipsoid reference plane (AUROC: 0.988, sensitivity: 93%, specificity: 96%) [33].

This study had certain limitations. This study was conducted retrospectively and with a relatively small sample size. Second, all study participants were Koreans, limiting the generalizability of the findings. Differences in the prevalence of keratoconus by race have been reported [11,34]. This difference in incidence is thought to affect differences in disease severity. Therefore, applying the present results to other ethnicities should be cautiously undertaken.

## 5. Conclusions

To conclude, this is the first study to investigate the diagnostic accuracy and clinical validation of the SCORE Analyzer for keratoconus using ANTERION, a new keratoconus screening program, in an Asian population. The SCORE Analyzer was found to be valid and consistent in Asian eyes, showing good sensitivity and specificity in keratoconus detection. We have successfully provided reference values for keratoconus diagnosis in the Asian population for the first time using SCORE Analyzer. We propose that the new SCORE Analyzer of ANTERION can be used as a useful indicator in the diagnosis of keratoconus in an Asian population.

## Figures and Tables

**Table 1:** Patient demographics of three groups.

Variables	Normal (*n* = 46)	KC Suspect (*n* = 45)	KC (*n* = 60)	*p* Value
**Age (years)**	29.76 ± 10.26	27.87 ± 10.43	30.28 ± 8.29	0.055 *
**Sex**				
Male:Female (*n*)	22:24	24:21	33:27	0.754 ^†^
**Laterality**				
Right	21 (45.65%)	26 (57.78%)	28 (46.67%)	0.428 ^†^
Left	25 (54.35%)	19 (42.22%)	32 (53.33%)	
**Refractive errors (D)**				
Spherical	−3.28 ± 2.41	−3.10 ± 2.71	−3.08 ± 3.04	0.892 *
Cylindrical	−0.94 ± 0.85	−2.07 ± 1.75	−2.67 ± 1.74	<0.001 *
**Average ant. Sim K (D)**	43.29 ± 1.73	44.48 ± 1.26	47.67± 5.71	<0.001
Steep (D)	44.13 ± 1.96	45.74 ± 1.68	49.55 ± 6.61	<0.001
Flat (D)	42.50 ± 1.66	43.32 ± 1.23	45.99 ± 5.19	<0.001
**Ant. astigmatism (D)**	1.62 ± 1.05	2.43 ± 1.49	3.56 ± 2.10	<0.001
**Ant. K max (D)**	44.71 ± 2.01	46.52 ± 1.68	53.95 ± 9.77	<0.001
**Average Post. K (D)**	−6.18 ± 0.28	−6.37 ± 0.23	−7.12 ± 1.20	<0.001
Steep (D)	−6.41 ± 0.34	−6.63 ± 0.30	−7.51 ± 1.29	<0.001
Flat (D)	−5.97 ± 0.25	−6.14 ± 0.22	−6.78 ± 1.16	<0.001
**Post. astigmatism (D)**	−0.44 ± 0.18	−0.49 ± 0.20	−0.73 ± 0.42	<0.001
**Post. K max (D)**	−6.48 ± 0.35	−6.76 ± 0.32	−8.86 ± 2.36	<0.001
**Average total K (D)**	42.74 ± 1.76	44.01 ± 1.34	47.55 ± 6.39	<0.001
Steep (D)	43.49 ± 1.98	45.18 ± 1.79	49.32 ± 7.19	<0.001
Flat (D)	42.00 ± 1.68	42.84 ± 1.23	45.75 ± 4.14	<0.001
Total K (D)	1.49 ± 1.02	2.34 ± 1.50	3.54 ± 2.46	<0.001
**Central pachymetry (µm)**	539.74 ± 32.07	537.04 ± 25.38	493.15 ± 50.38	<0.001
**Thinnest pachymetry (µm)**	537.11 ± 31.56	533.13 ± 25.23	477.03 ± 53.37	<0.001
**Thinnest point X**	0.01 ± 0.40	−0.07 ± 0.50	0.06 ± 0.61	0.358
**Thinnest point Y**	−0.38 ± 0.23	−0.46 ± 0.31	−0.72 ± 0.42	<0.001
**WTW**	12.22 ± 0.50	11.82 ± 0.54	12.08 ± 0.51	0.004

KC, keratoconus; D, diopter; WTW, white to white diameter; * Kruskal–Wallis test. *p* < 0.05 is statistically significant; ^†^ Chi-square test. *p* < 0.05 is statistically significant.

**Table 2:** SCORE parameters of three groups. (*p* < 0.05 is statistically significant).

SCORE Parameters	Normal (*n* = 46)	KC Suspect (*n* = 45)	KC (*n* = 60)	*p* Value *	*p* Value ^†^	*p* Value ^‡^	*p* Value ^§^
Total value	−0.55 ± 1.38 (−3.10, 3.00)	0.77 ± 2.10 (−1.90, 11.20)	14.43 ± 13.41 (−0.30, 72.10)	**<0.001**	**<0.001**	**<0.001**	**<0.001**
Ant. K max	44.58 ± 1.97 (41.20, 49.10)	46.48 ± 1.68 (42.75, 50.83)	53.96 ± 9.41 (44.34, 94.13)	**<0.001**	**<0.001**	**<0.001**	**<0.001**
AntK_oppoK	0.87 ± 0.74 (0.00, 2.64)	1.32 ± 1.01 (0.02, 4.66)	5.90 ± 4.76 (0.25, 16.89)	**<0.001**	**0.035**	**<0.001**	**<0.001**
Ant_inf_supK	0.26 ± 0.51 (−1.07, 1.36)	0.59 ± 0.80 (−1.38, 2.59)	4.37 ± 3.93 (−1.55, 20.69)	**<0.001**	**0.006**	**<0.001**	**<0.001**
Ant_irre_3 mm	0.91 ± 0.38 (0.31, 1.85)	1.27 ± 0.59 (0.29, 2.74)	2.80 ± 1.34 (0.60, 6.07)	**<0.001**	**0.007**	**<0.001**	**<0.001**
post_ele_thin	5.61 ± 4.07 (−1.00, 18.00)	10.33 ± 7.27 (2.00, 50.00)	52.82 ± 38.85 (9.00, 198.00)	**<0.001**	**<0.001**	**<0.001**	**<0.001**
A_Cor_thin	538.13 ± 34.35 (458, 596)	533.00 ± 25.10 (466, 578)	477.82 ± 53.17 (318, 568)	**<0.001**	0.319	**<0.001**	**<0.001**

KC: keratoconus; “Ant. K max”: The maximum K value of the anterior corneal surface; “Ant. Kmax—opposite K (AntK_oppoK)”: The difference between the maximum K value of the anterior corneal surface and the K value measured opposite the Kmax position; “Ant. inf.—sup. K mean (Ant_inf_supK)”: The difference between the inferior mean anterior corneal axial curvature and the superior mean anterior corneal axial curvature; “Ant. irregularity (Ant_irre_3 mm)”: The irregularity of the axial curvature of the anterior corneal surface at the central 3 mm ring; “Posterior elevation of thinnest point (post_ele_thin)”: The elevation of the posterior corneal surface at the location of the thinnest point with the best fit sphere as a reference surface; “Thinnest point(A_Cor_thin)”: The thinnest point of the cornea; * Kruskal–Wallis test; ^†^ Normal and KC suspect (Mann–Whitney test); ^‡^ Normal and KC (Mann–Whitney test); ^§^ KC suspect and KC (Mann–Whitney test).

**Table 3:** Stepwise multivariate linear regression analysis for SCORE analyzer with six parameters as the dependent variable.

	Parameters	Partial Regression Coefficient (B)	Standardized Partial Regression Coefficient (β)	*p*-Value
SCORE Value		(R = 0.991, R^2^ = 0.980, Adjusted R^2^ = 0.981)		
	Ant. K_max	0.344	0.229	<0.001
	AntK_oppoK	0.880	0.308	<0.001
	Ant_inf_supK	1.082	0.312	<0.001
	Ant_irre_3 mm	−0.939	−0.106	<0.001
	post_ele_thin	0.090	0.269	<0.001
	A_Cor_thin	−0.014	−0.063	0.001
	Constant	−9.267		0.003

SCORE, Screening Corneal Objective Risk of Ectasia; “Ant. Kmax”: The maximum K value of the anterior corneal surface; “Ant. Kmax—opposite K (AntK_oppoK)”: The difference between the maximum K value of the anterior corneal surface and the K value measured opposite the Kmax position; “Ant. inf.—sup. K mean (Ant_inf_supK)”: The difference between the inferior mean anterior corneal axial curvature and the superior mean anterior corneal axial curvature; “Ant. irregularity (Ant_irre_3 mm)”: The irregularity of the axial curvature of the anterior corneal surface at the central 3 mm ring; “Posterior elevation of thinnest point (post_ele_thin)”: The elevation of the posterior corneal surface at the location of the thinnest point with the best fit sphere as a reference surface; “Thinnest point (A_Cor_thin)”: The thinnest point of the cornea.

**Table 4:** Cut-off point and specificity and sensitivity values for each parameter tested in the normal, keratoconus, and keratoconus suspect groups.

	Parameters	AUROC	SE	*p* Value	Cut-Off	Sensitivity	Specificity	PLR	NLR
**KC vs. normal**	SCORE	0.974	0.012	0.000	2.40	0.868	0.978	39.97	0.14
	Ant. K max	0.934	0.024	0.000	45.80	0.950	0.804	4.86	0.06
	AntK_oppoK	0.917	0.026	0.000	2.67	0.733	1.000	-	0.27
	Ant_inf_supK	0.944	0.024	0.000	0.98	0.900	0.957	20.70	0.10
	Ant_irre_3 mm	0.932	0.024	0.000	1.41	0.867	0.935	13.29	0.14
	post_ele_thin	0.985	0.008	0.000	12.50	0.900	0.957	20.70	0.10
	A_Cor_thin	0.847	0.038	0.000	529.5	0.867	0.717	30.07	0.19
**KC suspect vs. Normal**	SCORE	0.729	0.053	0.000	−0.05	0.667	0.717	2.36	0.46
	Ant. K max	0.781	0.050	0.000	44.96	0.867	0.674	2.66	0.20
	AntK_oppoK	0.629	0.060	0.035	1.045	0.644	0.696	2.12	0.51
	Ant_inf_supK	0.667	0.059	0.006	0.90	0.40	0.957	9.20	0.63
	Ant_irre_3 mm	0.663	0.057	0.007	0.87	0.822	0.478	1.58	0.37
	post_ele_thin	0.754	0.050	0.000	10.5	0.511	0.913	5.88	0.54
	A_Cor_thin	0.561	0.061	0.319	547.5	0.756	0.435	1.34	0.56
**KC suspect vs. KC**	SCORE	0.923	0.026	0.000	4.45	0.817	0.978	36.75	0.19
	Ant. K max	0.865	0.035	0.000	48.275	0.717	0.911	8.06	0.31
	AntK_oppoK	0.859	0.036	0.000	3.075	0.700	0.978	31.50	0.31
	Ant_inf_supK	0.899	0.031	0.000	1.52	0.783	0.956	17.63	0.23
	Ant_irre_3 mm	0.858	0.036	0.000	1.655	0.800	0.800	4.00	0.25
	post_ele_thin	0.931	0.024	0.000	16.5	0.833	0.956	18.75	0.17
	A_Cor_thin	0.836	0.038	0.000	516	0.750	0.756	3.07	0.33
**KC suspect + KC vs. Normal**	SCORE	0.869	0.029	0.000	0.25	0.819	0.783	3.77	0.23
	Ant. K max	0.869	0.031	0.000	45.595	0.857	0.804	4.38	0.18
	AntK_oppoK	0.793	0.036	0.000	1.35	0.686	0.804	3.50	0.39
	Ant_inf_supK	0.825	0.033	0.000	0.90	0.686	0.957	15.77	0.33
	Ant_irre_3 mm	0.817	0.034	0.000	1.38	0.619	0.935	9.49	0.41
	post_ele_thin	0.886	0.026	0.000	10.5	0.743	0.913	8,54	0.28
	A_Cor_thin	0.724	0.045	0.000	529.5	0.657	0.717	2.33	0.48

AUROC, area under the receiver operating characteristic curve; KC, keratoconus PLR, positive likelihood ratio; NLR, negative likelihood ratio; “Ant. Kmax”: The maximum K value of the anterior corneal surface; “Ant. Kmax—opposite K (AntK_oppoK)”: The difference between the maximum K value of the anterior corneal surface and the K value measured opposite the Kmax position; “Ant. inf.—sup. K mean (Ant_inf_supK)”: The difference between the inferior mean anterior corneal axial curvature and the superior mean anterior corneal axial curvature; “Ant. irregularity (Ant_irre_3 mm)”: The irregularity of the axial curvature of the anterior corneal surface at the central 3 mm ring; “Posterior elevation of thinnest point (post_ele_thin)”: The elevation of the posterior corneal surface at the location of the thinnest point with the best fit sphere as a reference surface; “Thinnest point (A_Cor_thin)”: The thinnest point of the cornea.

## Data Availability

Research data are available through the corresponding author upon reasonable request.
